# Lactulose, Rifaximin, and Survival in Hepatic Encephalopathy: A Cohort Study of 120 Patients

**DOI:** 10.3390/jcm14207331

**Published:** 2025-10-17

**Authors:** Luana Alexandrescu, Ionut Tiberiu Tofolean, Cristina Aftenie, Daria Maria Alexandrescu, Doina Ecaterina Tofolean, Alina Doina Nicoara, Alina Mihaela Stanigut, Andreea Nelson Twakor, Elena Rusu, Eugen Dumitru, Andrei Dumitru, Cristina Tocia, Alexandra Herlo, Miruna Alexa Mihu, Ioana Popescu, Elena Dina, Bogdan Cimpineanu

**Affiliations:** 1 Gastroenterology Department, “Sf. Apostol Andrei” Emergency County Hospital, 145 Tomis Blvd., 900591 Constanta, Romania; alexandrescu_l@yahoo.com (L.A.); afteniecristina@gmail.com (C.A.); eugen.dumitru@yahoo.com (E.D.); dr.andreidumitru@gmail.com (A.D.); cristina.tocia@yahoo.com (C.T.); mihumiruna@yahoo.com (M.A.M.); ioanapop122@gmail.com (I.P.); elena.dina@ymail.com (E.D.); 2 Medicine Faculty, “Ovidius” University of Constanta, 1 Universitatii Street, 900470 Constanta, Romania; tofoleandoina@yahoo.com (D.E.T.); alina.stanigut@365.univ-ovidius.ro (A.M.S.); cimpineanub@yahoo.com (B.C.); 3 Faculty of Medicine, Titu Maiorescu University, 040051 Bucharest, Romania; alexandrescu_daria@yahoo.com (D.M.A.); elenarusu98@yahoo.com (E.R.); 4 Pneumology Department, “Sf. Apostol Andrei” Emergency County Hospital, 145 Tomis Blvd., 900591 Constanta, Romania; 5 Internal Medicine Department, “Sf. Apostol Andrei” Emergency County Hospital, 145 Tomis Blvd., 900591 Constanta, Romania; alina.nicoara@365.univ-ovidius.ro (A.D.N.); andreea.purcaru@365.univ-ovidius.ro (A.N.T.); 6 Nephrology Department, “Sf. Apostol Andrei” Emergency County Hospital, 145 Tomis Blvd., 900591 Constanta, Romania; 7 Department XIII, Discipline of Infectious Diseases, “Victor Babes” University of Medicine and Pharmacy Timisoara, 2 Eftimie Murgu Square, 300041 Timisoara, Romania; alexandra.mocanu@umft.ro

**Keywords:** hepatic encephalopathy, lactulose, rifaximin, cirrhosis, survival analysis, liver failure

## Abstract

**Background:** Hepatic encephalopathy (HE) is a severe neuropsychiatric complication of advanced liver disease, driven primarily by ammonia accumulation due to impaired hepatic detoxification and portosystemic shunting. Lactulose is a cornerstone therapy, while rifaximin serves as an effective adjunct for reducing recurrence and hospitalizations. **Methods:** We conducted a retrospective cohort study at Constanța Emergency County Hospital from January 2022 to March 2025, including 120 adult patients diagnosed with HE. Inclusion criteria were confirmed diagnosis of cirrhosis with clinical and/or biochemical evidence of HE. Patients with other primary neurological disorders or incomplete medical records were excluded. Data on demographics, comorbidities, laboratory results, and medications were collected. Statistical analyses were performed employing descriptive statistics, Friedman’s two-way ANOVA by ranks for medication use, and Cox proportional hazards regression to assess survival associations. **Results:** The mean age was 61.19 years, with high prevalence of anemia (mean hemoglobin: 9.35 g/dL) and thrombocytopenia (mean: 121.86 × 10^3^/µL). Inflammatory markers were elevated (mean CRP: 36.95 mg/L; ESR: 55.83 mm/h), and INR averaged 1.86. Lactulose was administered to 63.3% of patients, rifaximin to 52.5%, with diuretics, pantoprazole, and albumin being frequently used. Friedman’s analysis ranked lactulose highest in usage frequency. Cox regression indicated no significant short-term survival difference associated with toxic encephalopathy or rifaximin use. **Conclusion:** In this cohort, lactulose remained the primary treatment for HE, often complemented by supportive pharmacotherapy. While rifaximin use was limited, the overall medication patterns reflected standard practice priorities in HE management.

## 1. Introduction

Hepatic encephalopathy is a complex neuropsychiatric syndrome resulting from liver dysfunction that disrupts cerebral function via accumulation of neurotoxins, especially ammonia, due to both impaired hepatic detoxification and portosystemic shunting [1]. Among patients with cirrhosis, HE remains a leading cause of recurrent hospitalizations and mortality, and its management is critical in reducing both morbidity and healthcare burden [2]. According to the International Society for Hepatic Encephalopathy and Nitrogen Metabolism, minimum hepatic encephalopathy, which affects up to 80% of cirrhosis patients, has subtle symptoms only identifiable by specialized tests [3]. Hepatic encephalopathy causes confusion, personality changes, disorientation, and loss of consciousness.

The cornerstone of HE treatment involves reducing ammonia production and absorption through modulation of the gut [2]. Lactulose, a non-absorbable disaccharide, remains first-line therapy in most guidelines. It works by acidifying the colonic lumen, promoting conversion of ammonia (NH_3_) into the less absorbable ammonium ion (NH_4_^+^), thereby facilitating fecal excretion and improving neurocognition [4]. Nonetheless, many patients with recurrent or refractory HE require adjunctive therapy to achieve optimal control.

**Figure 1:**
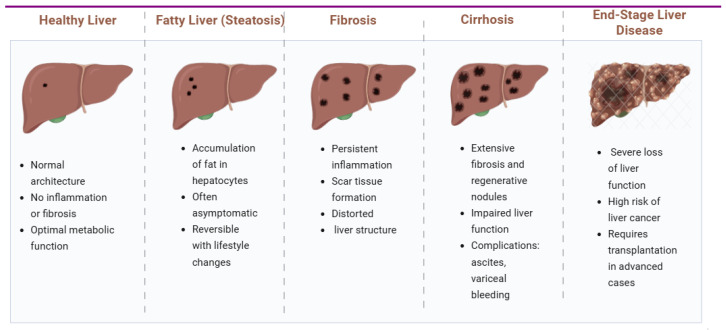
Stages of liver disease progression. Created with Biorender [5].

Liver disease progresses through distinct pathological stages, beginning with a healthy liver and advancing to end-stage disease if left untreated. Early alterations, such as fatty liver, are often reversible with lifestyle interventions [1]. Figure 1 shows how persistent liver injury may lead to fibrosis, characterized by scar tissue formation and structural distortion.

The final stage, end-stage liver disease, is associated with severe hepatic dysfunction and a heightened risk of hepatocellular carcinoma, often necessitating liver transplantation.

Rifaximin, a minimally absorbed oral antibiotic, targets ammonia-producing gut bacteria and reduces endotoxin levels [6]. Meta-analyses and randomized controlled trials confirm that combining rifaximin with lactulose significantly improves clinical efficacy, reduces recurrence of HE, decreases hospitalizations, and is associated with a mortality benefit compared to lactulose alone [7,8,9]. One pooled study including multiple trials specifically demonstrated that the rifaximin–lactulose combination significantly lowers the risk of HE recurrence and related hospital admissions over six months in cirrhotic patients with prior overt HE [6]. Yet, despite this strong evidence, rifaximin is often reserved as an add-on for patients inadequately responding to or intolerant of lactulose, partly due to cost considerations [10].

In Romania, there is a scarcity of real-world data regarding pharmacotherapy patterns and outcomes in HE. Specifically, it remains unclear how frequently these agents are used in routine clinical practice at tertiary centers, and what impact their use has on short-term prognosis. Therefore, this retrospective cohort study from Constanța Emergency County Hospital aims to analyze real-world utilization patterns of lactulose, rifaximin, and other critical care drugs in HE patients and evaluate their prognostic implications using advanced statistical modelling.

## 2. Materials and Methods

We conducted a retrospective observational cohort study on patients diagnosed with hepatic encephalopathy and admitted to Constanța Emergency County Hospital, Romania, between January 2022 and March 2025. The study was designed to assess the relationship between critical care drug administration, clinical parameters, and in-hospital prognosis. The final cohort comprised 120 patients. A formal a priori power calculation was not feasible given the retrospective design; however, the inclusion of all eligible cases during the study period provides a comprehensive representation of the patient population and minimizes selection bias. The absence of a sample size calculation is acknowledged as a limitation and further discussed in the Discussion section. The study was conducted in accordance with the Declaration of Helsinki, and approved by the Ethics Committee of “Sf. Apostol Andrei” Emergency County Hospital in Constanta, Romania, protocol no. 113/21.04.2025.

### 2.1. Patient Selection

Patients were identified through the hospital’s electronic medical record system. Diagnosis of hepatic encephalopathy was established according to clinical criteria (West Haven classification) and relevant laboratory/imaging findings.

For the purpose of this study, toxic encephalopathy was defined in accordance with the West Haven criteria for hepatic encephalopathy [11], which classify patients into grades I–IV based on alterations in consciousness, intellectual function, behavior, and neuromuscular activity. Only patients fulfilling these standardized diagnostic criteria were included.

### 2.2. Inclusion Criteria

- Age ≥ 18 years.
- Confirmed diagnosis of hepatic encephalopathy (grades I–IV).
- Documented underlying chronic liver disease (e.g., cirrhosis, chronic hepatitis, or toxic liver disease).
- Admission to Constanța Emergency County Hospital during the study period.
- Availability of complete clinical, laboratory, and treatment records.

### 2.3. Exclusion Criteria

- Acute liver failure without chronic liver disease background.
- Neurological disorders unrelated to hepatic encephalopathy (e.g., stroke, brain tumors).
- Severe psychiatric disorders precluding accurate HE assessment.
- Transfer to another medical facility before treatment completion.
- Missing or incomplete key variables for statistical analysis.

### 2.4. Data Collection

The following data were extracted from patient records:

- Demographic variables: age, sex.
- Clinical variables: etiology of liver disease, comorbidities, complications during hospitalization.
- Laboratory parameters: hematological profile, liver function tests, renal function, inflammatory markers, coagulation profile, and tumor markers when available.
- Treatment variables: administration of lactulose, rifaximin, diuretics, vasoactive agents, antibiotics, and other supportive drugs.
- Outcome measures: in-hospital mortality, days of hospitalization, presence of toxic encephalopathy.

### 2.5. Statistical Analysis

Data were analyzed using IBM SPSS Statistics version 29.0 (IBM Corp., Armonk, NY, USA). Continuous variables were expressed as mean ± standard deviation (SD) or median (interquartile range, IQR) depending on the distribution assessed by the Shapiro–Wilk test [12]. Categorical variables were expressed as counts and percentages.

Comparisons between groups were made using:

- Independent-samples t-test or Mann–Whitney U test for continuous variables.
- Chi-square test or Fisher’s exact test for categorical variables.

To evaluate differences in the relative frequency of multiple related treatments, we applied the related-samples Friedman’s two-way analysis of variance by ranks [13]. Survival analysis was performed using the Cox proportional hazards regression model, reporting hazard ratios (HR) with 95% confidence intervals (CI), and adjusting for covariates including toxic encephalopathy status. A *p*-value < 0.05 was considered statistically significant [14].

## 3. Results

Table 1 summarizes the baseline demographic and clinical features of the 120 patients included in the study, all diagnosed with hepatic encephalopathy.

The majority of the cohort were male (67.5%) and two-thirds had underlying chronic liver failure. Alcoholic liver cirrhosis was identified in 17.5% of patients, while other forms of cirrhosis and toxic liver disease with fibrosis and cirrhosis accounted for 26.7% and 25.8% of cases, respectively. Notably, esophageal varices were present in 40% of patients, and jaundice was observed in over half (58.3%). Hypersplenism and thrombocytopenia were prevalent, affecting approximately one-third and more than half of the cohort, respectively.

Comorbid conditions were common, with essential hypertension present in 24.2% and diabetes mellitus in 50.8% of patients. Portal hypertension was identified in a quarter of the cohort, while anemia (both recorded as diagnosis 1 and unclassified) was noted in over half. Complications related to liver disease were frequent, including ascites (78.3%), gastrointestinal hemorrhage (13.3%), hematemesis (18.3%), and acute respiratory failure (2.5%). Less frequent but clinically significant findings included Clostridium difficile enterocolitis, urinary tract infections, and mental or behavioral disorders.

Table 2 below presents the main characteristics of the patients included in the study.

Notably, the cohort exhibited a wide age distribution (35–82 years), underlining the heterogeneity of HE presentation across different age groups. The variability in liver enzymes was striking, with ALT ranging from 10 to 3148 U/L and AST from 4 to 4167 U/L, reflecting the diverse severity of hepatic injury. Similarly, total bilirubin values ranged from normal to 29 mg/dL, again highlighting the clinical heterogeneity of the patient sample.

Hemoglobin levels averaged 9.35 g/dL, while platelet counts were substantially reduced (mean: 121.86 × 10^3^/µL), consistent with hypersplenism and portal hypertension. Inflammatory markers such as C-reactive protein (mean: 36.95 mg/L) and ESR (mean: 55.83 mm/h) were elevated. Liver enzyme levels (ALT, AST, and GGT) displayed high standard deviations. Markers of synthetic liver function revealed compromised hepatic reserve, with mean INR of 1.86 and fibrinogen levels averaging 2.26 g/L. Total bilirubin levels were elevated in many cases (mean: 4.73 mg/dL), while hypoalbuminemia was reflected in total serum protein measurements. Renal function parameters showed mild elevations in urea and creatinine. Tumor markers such as AFP, CEA, and CA 19-9 were assessed in selected cases, with variable results.

In Table 3 we present the spectrum and frequency of pharmacological agents administered to patients in our study cohort.

Values are expressed as absolute number of patients (N) and percentage of the total cohort. Only core medications directly related to HE management or cirrhosis complications are included. Other supportive therapies are presented in Supplementary Table S1.

Antibiotic use was common, with rifaximin (52.5%), ceftriaxone (40.0%), and ceftamil (3.3%) representing agents from different antimicrobial classes. Rifaximin was the most frequently prescribed medication (82/5%) in this category, reflecting its pivotal role in the management of hepatic encephalopathy by reducing ammonia-producing gut flora. Other broad-spectrum antibiotics, such as meropenem (11.7%) and vancomycin (6.7%), were administered less frequently. Diuretic therapy was widely utilized, with furosemide (53.3%), spironolactone (45.8%), and diurex (39.2%) being prescribed to manage ascites and volume overload associated with portal hypertension. Gastroprotective agents like pantoprazole (50.8%) were also common, alongside lactulose (63.3%) for ammonia reduction and constipation management. Vitamin supplementation, including vitamin B1 (23.3%) and vitamin B6 (21.7%), addressed nutritional deficiencies frequently seen in chronic liver disease. Symptomatic relief medications such as algocalmin (33.3%) and diazepam (7.5%) were used selectively to manage pain or agitation.

Several drugs in the table indicate acute care interventions. Vasoactive agents such as noradrenaline (41.7%) and adrenaline (1.7%) were administered in hemodynamic instability, while dobutamine (1.7%) was reserved for cardiac support. Albumin infusions (Albutein, 38.3%) were frequently employed for volume expansion, particularly in paracentesis or spontaneous bacterial peritonitis. Hydrocortisone (9.2%) and sandostatin (20.0%) were used for refractory shock or variceal bleeding control.

**Figure 2:**
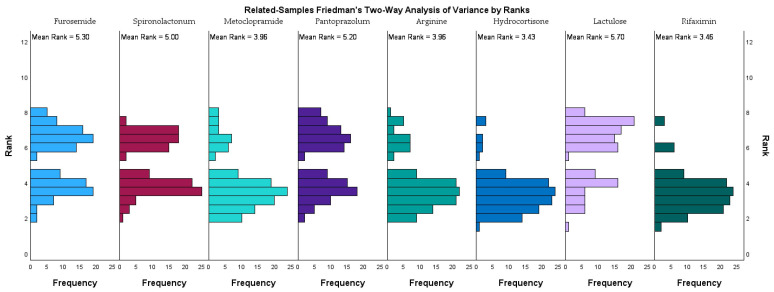
Related-samples Friedman’s two-way analysis of variance by ranks for selected medications in our study cohort.

Figure 2 illustrates the rank distribution and mean ranks of eight frequently administered medications in the study cohort, as analyzed using the related-samples Friedman’s two-way analysis of variance by ranks.

Lactulose had the highest mean rank (5.36), followed closely by rifaximin (5.33), furosemide (4.96), and pantoprazole (4.86), indicating that these medications were among the most consistently administered across the study population. Spironolactone also ranked highly (4.66), reflecting its frequent use in managing fluid retention. In contrast, arginine (3.63) and metoclopramide (3.63) had lower mean ranks, while hydrocortisone recorded the lowest mean rank (3.09), suggesting a more selective or situational use. These ranking trends align with therapeutic priorities in managing hepatic encephalopathy within advanced liver disease. The prominence of lactulose and rifaximin stresses their established synergistic role in reducing ammonia levels and improving neurocognitive outcomes. The frequent use of diuretics such as furosemide and spironolactone highlights the need for effective ascites and volume management.

We performed a Cox proportional hazards regression to evaluate the association between medication use and short-term survival. In this model, lactulose use was not significantly associated with improved survival (HR = 1.042, 95% CI: 0.667–1.628, *p* = 0.856), consistent with its nearly universal prescription across patients irrespective of outcome. Rifaximin use also did not show a statistically significant survival advantage (HR = 0.928, 95% CI: 0.605–1.423, *p* = 0.732); however, interpretation is limited by sample size and the retrospective design. The absence of censored cases further reduces the reliability of hazard ratio estimates, and thus these findings should be regarded as descriptive rather than conclusive. Tables S2 and S3 include the event censoring distribution.

In the Cox regression model, patients were categorized according to whether they received lactulose (76 patients, 63.3%) or not (44 patients, 36.7%), and whether they received rifaximin (63 patients, 52.5%) or not (57 patients, 47.5%). These categories were used to examine potential associations with short-term survival.

**Figure 3:**
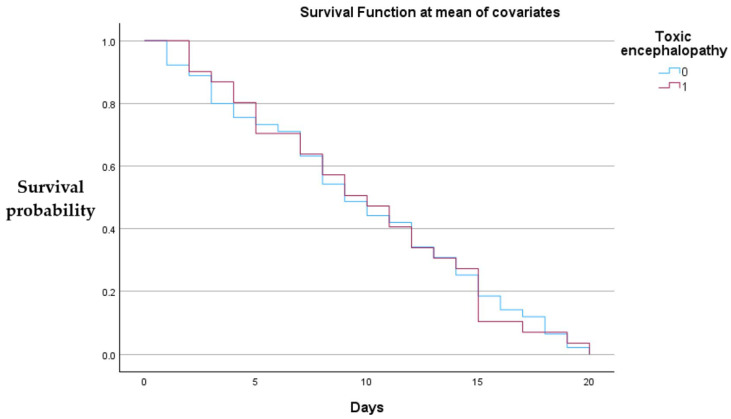
Cox proportional hazards survival curves for patients with and without toxic encephalopathy.

Figure 3 below shows that neither lactulose nor rifaximin administration showed a statistically significant effect.

Both groups demonstrate a gradual decline in survival over the 20-day observation period, with no evident large divergence between the curves, suggesting that toxic encephalopathy status did not produce a substantial difference in short-term survival when other covariates were held constant.

**Figure 4:**
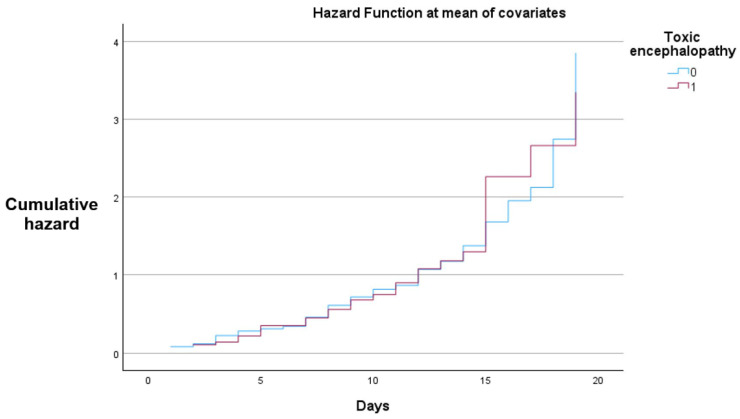
Cox proportional hazards cumulative hazard curves for patients with and without toxic encephalopathy.

Figure 4 demonstrates a higher cumulative hazard for the patients with toxic encephalopathy.

In the Cox proportional hazards model, all patients in both strata experienced the event (death) during the observation period, with no censored cases. While hazard ratios were formally calculated, the absence of censoring substantially limits their interpretability, and these results should be viewed with caution.

Both groups demonstrate a progressive increase in cumulative hazard over the 20-day follow-up, with closely aligned trajectories throughout most of the observation period. Slight divergence is observed in the later stages, where the toxic encephalopathy group shows a marginally higher cumulative hazard.

**Figure 5:**
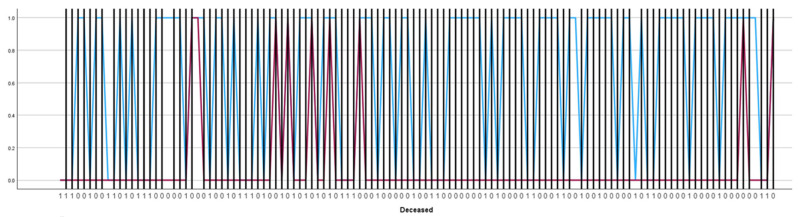
Distribution of Lactulose and Rifaximin use in relation to in-hospital mortality in patients with hepatic encephalopathy.

Figure 5 displays the binary distribution of lactulose (blue) and rifaximin (red) administration among the 120 patients in the study cohort, stratified by mortality status (Deceased = 1, Survived = 0). Each vertical bar represents an individual patient, with the y-axis indicating the presence (1) or absence (0) of each medication. The x-axis is ordered by patient mortality, allowing direct visual comparison between survivors and non-survivors.

Figure 5 shows that lactulose was prescribed to nearly two-thirds of the cohort in both survivors and non-survivors, confirming its role as a standard first-line therapy in hepatic encephalopathy. By contrast, rifaximin was prescribed in just over half of patients, typically those with more severe or recurrent episodes, reflecting its position as a selective adjunctive therapy in real-world practice. Importantly, the distribution of both drugs across survival outcomes indicates that treatment allocation followed established clinical priorities but was not strongly discriminatory for short-term survival, likely due to the advanced disease stage of most patients.

Due to the retrospective nature of data collection, it was not possible to reliably distinguish patients who received simultaneous combination therapy with lactulose and rifaximin from those who received monotherapy. Therefore, combination therapy could not be analyzed as a separate subgroup in this study.

From a clinical perspective, the high prevalence of lactulose use reflects its established role as first-line therapy for hepatic encephalopathy, regardless of patient prognosis. The relatively low rifaximin usage may be influenced by treatment protocols prioritizing it as an adjunct for refractory cases or secondary prevention, as well as potential cost or availability factors. From a clinical perspective, lactulose use was nearly universal across both survivors and non-survivors, confirming its role as the cornerstone therapy for hepatic encephalopathy regardless of outcome. Rifaximin, by contrast, was prescribed selectively, often in patients with recurrent or more severe presentations, reflecting both its established role as an adjunct therapy and its limited availability in our setting. Thus, clinical practice remains driven by guideline-based treatment principles, while the statistical models serve only to illustrate usage distributions and do not override the clinical interpretation.

Majority of patients with hepatic encephalopathy presented with advanced liver disease, frequent comorbidities, and significant laboratory abnormalities, particularly anemia, thrombocytopenia, and elevated inflammatory and liver injury markers. Lactulose emerged as the most consistently administered medication, underscoring its role as the cornerstone of treatment, while rifaximin was used selectively, often as an adjunct therapy. Friedman’s analysis ranked lactulose, diuretics, and pantoprazole highest in relative frequency of use, reflecting therapeutic priorities in managing hepatic encephalopathy and its complications. Cox proportional hazards modeling indicated no substantial short-term survival difference between patients with and without toxic encephalopathy, and the distribution of lactulose and rifaximin use across mortality outcomes suggested that these treatments were not strongly discriminatory for survival within the study period.

Because of the retrospective nature and limited sample size, we were unable to stratify patients into homogeneous subgroups by age, biochemical parameters, or specific adjunctive treatments such as ursodeoxycholic acid or essential phospholipids. This limited our ability to detect potential subgroup-specific associations.

## 4. Discussion

To our knowledge, this is the first real-world analysis of hepatic encephalopathy management and short-term survival from a Romanian tertiary care center. This adds novelty to the current literature, as most available real-life studies originate from Western Europe and North America. Our findings therefore provide valuable insight into local practice patterns, resource limitations, and their potential impact on outcomes. We compared our mortality data with those of international real-life studies reporting higher rifaximin use. In our cohort, rifaximin was prescribed to 52.5% of patients, a proportion comparable to some international reports but still lower than the 80–90% lactulose and 30–50% rifaximin usage rates frequently documented in large registry-based analyses and systematic reviews [6,15,16].

This pattern reflects current clinical guidelines emphasizing lactulose as first-line therapy, with rifaximin reserved for selected cases due to cost and availability considerations.

Our results align with those of Fu et al. [15] who demonstrated that combination therapy with lactulose and rifaximin significantly improved clinical efficacy and reduced recurrence rates in HE. Although rifaximin was less prescribed in our cohort, the principle of targeting ammonia production and absorption via both nonabsorbable disaccharides and antibiotics remains central to HE management in both settings. Similarly, Abraldes et al. [16] highlighted lactulose as the therapeutic cornerstone, with rifaximin as an adjunct to prevent recurrent episodes, mirroring the hierarchy of treatment we observed.

The low rifaximin use in our study may partially explain the absence of statistically significant survival differences in our Cox regression analysis. In contrast, Singh et al. [17] reported improved long-term outcomes and reduced hospitalization rates when rifaximin was used consistently as an adjunct to lactulose.

Regarding comorbidities, our cohort had a high prevalence of anemia, thrombocytopenia, and portal hypertension, findings that are consistent with those reported by Frenette et al. [18] who examined HE-related hospitalizations in the context of decompensated cirrhosis. Elevated inflammatory markers in our patients further support the systemic inflammatory component of HE pathophysiology described in their work.

An important limitation of our study is the inability to isolate and analyze patients on combination therapy (lactulose + rifaximin) separately. Although combination therapy is the most effective regimen for recurrent hepatic encephalopathy, our dataset did not allow for this level of stratification. This gap highlights the need for future prospective studies to evaluate combination therapy in real-world settings with adequate documentation.

Our patient population’s demographic and clinical profile also resonates with Liang et al. [19] who emphasized the role of primary prevention strategies in high-risk cirrhotic patients. While our study was retrospective and focused on in-hospital management, the high recurrence risk seen in our population suggests that preventive measures, such as earlier rifaximin initiation, could be valuable.

When comparing our findings to international cohorts, notable differences in medication usage patterns emerge. In our study, lactulose was administered to 63.3% of patients, whereas large registry-based analyses and systematic reviews from Western Europe and North America report usage rates exceeding 80–90% [6,15]. Similarly, rifaximin was prescribed to 52.5% of our cohort, a comparable percentage to international studies where adjunctive rifaximin therapy is used in 30–50% of patients with recurrent hepatic encephalopathy [7,17]. This discrepancy likely reflects differences in healthcare resources, reimbursement policies, and national guideline implementation. In terms of outcomes, while international data suggest reduced recurrence and hospitalization rates with combined rifaximin–lactulose therapy, our limited use of rifaximin may partially explain the absence of a survival benefit in the present cohort.

From a pharmacologic standpoint, Friedman’s two-way ANOVA by ranks highlighted lactulose, pantoprazole, and diuretics among the most frequently administered medications. This reflects not only HE management priorities but also the need to address complications such as ascites, gastrointestinal bleeding, and acid suppression in critically ill patients. The combination of these therapeutic interventions parallels the multidrug regimens described in Abraldes et al. [16] and Singh et al. [17] where HE treatment is integrated into the broader context of decompensated cirrhosis management.

The impact of chronic liver disease etiology on HE outcomes warrants specific attention. In our cohort, patients with alcohol-related cirrhosis had a greater burden of complications, including ascites and gastrointestinal hemorrhage, which may have contributed to poorer short-term outcomes. In contrast, patients with viral or toxic cirrhosis frequently presented with laboratory features such as hyperbilirubinemia and cytopenias, reflecting differences in disease progression and systemic involvement. These findings are consistent with reports from international studies showing that cirrhosis etiology can shape the risk profile, recurrence rate, and prognosis of HE [18,19].

An important limitation of our study is the incomplete availability of certain laboratory variables, such as BNP and CA 19-9, which were recorded in only a small subset of patients. This limited sample size reduces the reliability of statistical interpretation for these parameters and may introduce bias. Therefore, the findings related to these variables should be considered exploratory rather than conclusive, and they warrant confirmation in larger prospective studies with more complete datasets.

Another limitation of our survival analysis is the lack of censored cases, as all patients experienced the event during the study period. This prevents robust interpretation of hazard ratios and restricts the ability to generalize survival outcomes beyond the short-term timeframe of our cohort. Our survival modeling did not demonstrate statistically significant differences between treatment groups, largely due to methodological constraints (absence of censored cases) and the short follow-up window. However, the clinical relevance remains clear: lactulose was the most consistently used therapy, while rifaximin was reserved for selected patients, aligning with international guideline hierarchies. Thus, while the statistical models provide supportive context, the main interpretation for clinicians is that our center mirrors real-world practice where lactulose dominates therapy, and rifaximin is underutilized compared to Western cohorts. We also note that while Cox proportional hazards regression did not demonstrate significant survival associations for lactulose or rifaximin, this result must be interpreted cautiously due to sample size limitations, the absence of censored cases, and the retrospective design. The statistical analysis supports our descriptive findings but does not replace their clinical interpretation.

Another key limitation is the heterogeneity of the study population. Patients differed substantially in age, laboratory values (including liver enzymes, bilirubin, and protein levels), and adjunctive treatments such as ursodeoxycholic acid or essential phospholipids. Given the retrospective design and incomplete availability of certain variables, we could not reliably divide patients into comparable subgroups. We acknowledge that such stratification might have yielded more nuanced results, and we recommend that future prospective studies with larger cohorts incorporate subgroup analyses to better delineate treatment effects across different clinical profiles.

Our findings are in line with international literature regarding lactulose’s central role in HE therapy, the potential benefits of combination regimens, and the importance of managing comorbid conditions. Future prospective studies in our center should assess the cost-effectiveness and clinical benefits of broader rifaximin use, particularly in patients with recurrent HE episodes.

## 5. Conclusions

This study provides a detailed analysis of pharmacologic management and outcomes in patients with hepatic encephalopathy. Lactulose emerged as the most frequently prescribed medication, in line with current international guidelines recommending it as first-line therapy. Rifaximin use was markedly limited, reflecting potential accessibility and cost constraints, which may have influenced recurrence prevention and long-term outcomes. The patient cohort exhibited a high burden of cirrhosis-related comorbidities, including portal hypertension, anemia, thrombocytopenia, and elevated inflammatory markers, underscoring the complex clinical profile of HE in advanced liver disease.

Statistical analyses demonstrated the predominance of lactulose and other supportive medications such as diuretics, proton pump inhibitors, and antibiotics in routine management. However, no significant survival differences were observed based on rifaximin use, likely due to its limited administration.

Overall, our findings reinforce the central role of lactulose in HE management while highlighting the potential benefits of expanding rifaximin availability to improve recurrence prevention. Future prospective studies are warranted to evaluate the impact of combination therapy on survival, hospitalization rates, and quality of life in this patient population, with consideration for cost-effectiveness and accessibility in clinical decision-making.

## Figures and Tables

**Table 1:** Baseline clinical and demographic characteristics of the study cohort.

	Male	Alcoholic Liver Cirrhosis	Other Cirrhosis	Toxic Liver Disease with Fibrosis and Cirrhosis	Chronic Liver Failure	Alcoholic Hepatitis	Chronic Viral Hepatitis B	Chronic Viral Hepatitis C	Esophageal Varices
Skewness	−0.757	1.732	1.069	1.118	−0.757	6.162	6.162	4.182	0.413
Kurtosis	−1.452	1.018	−0.873	−0.763	−1.452	36.582	36.582	15.751	−1.860
Total	81	21	32	31	81	3	3	6	48
	Jaundice	Alcohol consumption	Hypersplenism	Thrombocytopenia	Malignant liver tumor	Malignant tumor, site unspecified	Albumin abnormality	Acute respiratory failure	GI hemorrhage
Skewness	−0.342	0.798	0.757	−0.238	5.266	10.954	0.135	6.162	2.185
Kurtosis	−1.915	−1.386	−1.452	−1.977	26.161	120.000	−2.016	36.582	2.820
Total	70	38	39	67	4	1	56	3	16
	Hematemesis	Ascites	Acidosis	Unspecified encephalopathy	Toxic encephalopathy	Essential hypertension	Portal hypertension	Diabetes	Cholangitis
Skewness	1.658	−1.393	1.334	−1.069	1.169	1.896	1.222	−0.034	10.954
Kurtosis	0.760	−0.061	−0.225	−0.873	−0.644	1.620	−0.515	−2.033	120.000
Total	22	94	27	88	30	19	29	61	1
	Anemia	Unclassified hemorrhage	Hypo-osmolality and hyponatremia	Hypokalemia	Chronic kidney disease	C. difficile enterocolitis	Urinary tract infection	Other venous disorders	Mental disorders
Skewness	−0.272	10.954	0.757	1.277	4.646	5.266	2.866	10.954	5.266
Kurtosis	−1.959	120.000	−1.452	−0.376	19.913	26.161	6.320	120.000	26.161
Total	68	1	39	28	5	4	11	1	4

**Table 2:** Descriptive statistics for patients included in the study.

Descriptive Statistics					
	N	Minimum	Maximum	Mean	Std. Deviation
Age	120	35	82	61.19	10
Blood parameters					
Hemoglobin	65	2	17	9.35	3
White blood cells	63	2	32	9.66	6
Platelets	63	24	367	121.86	75
CK-MB	27	−13.27	56.58	21.20	18
Troponin	32	−0.28	1.24	0.43	0
BNP	11	20.57	403.44	211.25	127
Serum glucose	114	100	456	40,176.49	425
Glycated hemoglobin	120	0	1	0.02	0
ALT	82	10	3148	73.73	345
AST	83	4	4167	135.07	476
GGT	84	11	2090	134.65	255
Alkaline phosphatase	65	0.00	402.27	161.40	103
Amylase	84	0.00	237.7900	90.69	55
Lipase	68	0.00	311.1600	92.20	57
Total bilirubin	82	0	29	4.73	5
Direct bilirubin	104	0	24	3.08	4
Indirect bilirubin	92	0	10	1.49	2
CA 19-9	8	31.80	95.270	59.28	24
CEA	40	0.00	9.56	3.07	2
AFP	41	0.00	22.52	10.59	6
Uric acid	61	1.95	13.71	6.08	2
Urea	114	5	312	74.99	65
Creatinine	114	0	6	1.24	1
INR	112	1	4	1.86	1
aPTT	112	20.00	60.86	40.89	9
C-reactive protein	82	0.00	103.00	36.95	24
ESR	75	0.00	140.00	55.82	27
Fibrinogen	79	0.64	3.91	2.25	1
D-dimers	7	937.87	3570.43	2062.8129	819
Serum sodium	62	121.38	142.95	132.36	5
Serum potassium	65	2.80	6.46	4.30	1
Alkaline reserve	108	11.11	30.00	20.45	4
Total serum proteins	8	5.42	7.03	6.1687	0
LDH	48	120.00	613.49	329.90	126
Serum calcium	24	7.00	10.40	8.60	1

N = number of patients with available data for each parameter; Minimum and Maximum = observed range; Mean = average value; Std. Deviation = measure of variability. Laboratory parameters include hematologic, biochemical, and inflammatory markers relevant to hepatic encephalopathy. ALT = alanine aminotransferase; AST = aspartate aminotransferase; GGT = gamma-glutamyl transferase; INR = international normalized ratio; ESR = erythrocyte sedimentation rate; CRP = C-reactive protein.

**Table 3:** Distribution and frequency of the most relevant medications administered to patients with hepatic encephalopathy.

	Ceftazidime	Ceftriaxone	Cefort	Vancomycin	Ciprofloxacin	Meropenem	Metronidazole	Ampicillin	Cefuroxime
Skewness	5.266	0.413	1.985	3.519	3.817	2.419	3.817	2.701	3.817
Kurtosis	26.161	−1.860	1.974	10.556	12.781	3.914	12.781	5.382	12.781
Total	4	48	18	8	7	14	7	12	7
	Smecta	Furosemide	Diurex *	SPL	Paracetamol	UDCA	EPL	Lactulose	Rifaximin
Skewness	10.954	−0.135	0.450	0.169	3.817	0.342	4.182	−0.560	3.268
Kurtosis	120.000	−2.016	−1.829	−2.005	12.781	−1.915	15.751	−1.715	8.828
Total	1	64	47	55	7	50	6	76	63
	Calcium gluconate	Meto- clopramide	Panto- prazolum						
Skewness	1.222	1.519	−0.034						
Kurtosis	−0.515	0.312	−2.033						
Total	29	24	61						

* Diurex-Spironolactonum + Furosemide; SPL—Spironolactone; UDCA—Ursodeoxycholic Acid; EPL-Essential phospholipids.

## Data Availability

The data presented in this study are available on request from the corresponding author.

## Supplementary Materials

The following supporting information can be downloaded at: https://www.mdpi.com/article/10.3390/jcm14207331/s1, Table S1. Distribution and frequency of the most relevant medications administered to patients with hepatic encephalopathy; Table S2. Event and censoring distribution by toxic encephalopathy strata in the Cox proportional hazards model; Table S3. Coding of categorical variables (Lactulose and Rifaximin) for the Cox regression analysis.

## References

[1] Oriko D.O., Khawaj Z., Cheema M.U., Talreja A., Tayyab M.A., Zamir M.H., Iqbal M., Farooq U., Ekomwereren O., Tariq M.M. (2025). Therapeutic Duel of Rifaximin Versus Lactulose in Hepatic Encephalopathy: A Systematic Review. Cureus.

[2] Swansson W.D., Anderson B.M., Yeoh S.W., Lewis D.J. (2023). Management of minimal and overt hepatic encephalopathy with branched-chain amino acids: A review of the evidence. Eur. J. Gastroenterol. Hepatol..

[3] Jain A., Sharma B.C., Mahajan B., Srivastava S., Kumar A., Sachdeva S., Sonika U., Dalal A. (2022). L-ornithine L-aspartate in acute treatment of severe hepatic encephalopathy: A double-blind randomized controlled trial. Hepatology.

[4] Bloom P.P., Tapper E.B. (2023). Lactulose in cirrhosis: Current understanding of efficacy, mechanism, and practical considerations. Hepatol. Commun..

[5] BioRender.com Scientific Image and Illustration Software. https://www.biorender.com/.

[6] Caraceni P., Vargas V., Solà E., Alessandria C., de Wit K., Trebicka J., Angeli P., Mookerjee R.P., Durand F., Pose E. (2021). The use of rifaximin in patients with cirrhosis. Hepatology.

[7] Wang Z., Chu P., Wang W. (2018). Combination of rifaximin and lactulose improves clinical efficacy and mortality in patients with hepatic encephalopathy. Drug Des. Devel. Ther..

[8] American Association for the Study of Liver Diseases (AASLD) (2021). Why Do We Use Lactulose and Rifaximin for Hepatic Encephalopathy? Liver Fellow Network. https://www.aasld.org/liver-fellow-network/core-series/why-series/why-do-we-use-lactulose-and-rifaximin-hepatic.

[9] Rahimi R.S., Brown K.A., Flamm S.L., Brown R.S. (2021). Overt hepatic encephalopathy: Current pharmacologic treatments and improving clinical outcomes. Am. J. Med..

[10] Cochrane (2025). Rifaximin for Prevention and Treatment of Hepatic Encephalopathy in People with Cirrhosis. Cochrane. https://www.cochrane.org/evidence/CD011585_rifaximin-prevention-and-treatment-hepatic-encephalopathy-people-cirrhosis.

[11] Weissenborn K. (2019). Hepatic encephalopathy: Definition, clinical grading and diagnostic principles. Drugs.

[12] De Souza R.R., Toebe M., Mello A.C., Bittencourt K.C. (2022). Sample size and Shapiro-Wilk test: An analysis for soybean grain yield. Eur. J. Agron..

[13] McClenaghan E., McClenaghan E. The Friedman Test. https://www.technologynetworks.com/tn/articles/the-friedman-test-387454.

[14] Bradburn M.J., Clark T.G., Love S.B., Altman D.G. (2003). Survival analysis part II: Multivariate data analysis—An introduction to concepts and methods. Br. J. Cancer.

[15] Fu J., Gao Y., Shi L. (2022). Combination therapy with rifaximin and lactulose in hepatic encephalopathy: A systematic review and meta-analysis. PLoS ONE.

[16] Abraldes J.G., Caraceni P., Ghabril M., Garcia-Tsao G. (2023). Update in the Treatment of the Complications of Cirrhosis. Clin. Gastroenterol. Hepatol..

[17] Singh J., Ebaid M., Saab S. (2024). Advances in the management of complications from cirrhosis. Gastroenterol. Rep..

[18] Frenette C.T., Levy C., Saab S. (2022). Hepatic Encephalopathy-Related Hospitalizations in Cirrhosis: Transition of Care and Closing the Revolving Door. Dig. Dis. Sci..

[19] Liang A.B., Brar S.B., Almaghrabi M., Khan M.Q., Qumosani K., Teriaky A. (2023). Primary prevention of hepatic encephalopathy post-TIPS: A systematic review and meta-analysis. Medicine.

