# Supplementary material for: Lactulose, Rifaximin, and Survival in Hepatic Encephalopathy: A Cohort Study of 120 Patients
Source: J Clin Med. 2025 Oct 17;14(20):7331. doi: 10.3390/jcm14207331 (PMC12565197; doi:10.3390/jcm14207331)
Supplement: Supplementary file 1 [file jcm-14-07331-s001.zip › jcm-3840147-supplementary.pdf]

Table S1. Distribution and frequency of the most relevant medications administered to patients with hepatic encephalopathy.

	**Ceftazidime**	**Ceftriaxone**	**Cefort**	**Vancomycin**	**Ciprofloxacin**	**Meropenem**	**Metronidazole**	**Ampicillin**	**Cefuroxime**
Skewness	5.266	0.413	1.985	3.519	3.817	2.419	3.817	2.701	3.817
Kurtosis	26.161	-1.860	1.974	10.556	12.781	3.914	12.781	5.382	12.781
Total	4	48	18	8	7	14	7	12	7
	Smecta	Furosemide	Diurex*	SPL	Paracetamol	Vitamin B1	Vitamin B6	Algocalmin*	Diazepam
Skewness	10.954	-0.135	0.450	0.169	3.817	1.277	1.393	0.716	3.268
Kurtosis	120.000	-2.016	-1.829	-2.005	12.781	-0.376	-0.061	-1.513	8.828
Total	1	64	47	55	7	28	26	40	9
	Calcium gluconate	Meto-clopramide	No-Spa*	Panto-prazolum	Sonovue*	Acupan*	Albutein	Phyto-menadione	Adrenaline
Skewness	1.222	1.519	2.081	-0.034	3.817	6.162	0.486	1.519	1.334
Kurtosis	-0.515	0.312	2.371	-2.033	12.781	36.582	-1.794	0.312	-0.225
Total	29	24	17	61	7	3	46	24	27
	Dobutamine	Amlodipine	NAC	Arginine	GC	SG	Moxonidine	Octreotide	Noradrenaline
Skewness	7.647	7.647	1.021	1.519	3.519	1.021	10.954	1.519	2.866
Kurtosis	57.432	57.432	-0.975	0.312	10.556	-0.975	120.000	0.312	6.320
Total	2	2	33	24	8	33	1	24	11
	UDCA	EPL	Lactulose	Rifaximin					
Skewness	0.342	4.182	-0.560	3.268					
Kurtosis	-1.915	15.751	-1.715	8.828					
Total	50	6	76	63					

*Diurex-Spironolactonum+Furosemide; SPL – Spironolactone; Algocalmin - Metamizole sodium; No-Spa - Drotaverine hydrochloride; Sonovue - Sulfur hexafluoride; Acupan - Nefopam hydrochlorid; GC – Hydrocortisone; NAC – N-acetyl cysteine; SG - Succinylated Gelatin; UDCA-Ursodeoxycholic Acid; EPL-Essential phospholipids

Table S2. Event and censoring distribution by toxic encephalopathy strata in the Cox proportional hazards model.

Stratum Status[a]			
Stratum	Event	Censored	Censored Percent
0	90	0	0.0%
1	30	0	0.0%
Total	120	0	0.0%

a. The stratum variable is toxic encephalopathy.

Two strata were analyzed: stratum 0 (no toxic encephalopathy) and stratum 1 (presence of toxic encephalopathy). In both strata, all patients experienced the event (death) within the observation period, with zero censored cases, yielding a 0.0% censoring rate in each group. The total cohort included 120 patients, of whom 90 were in stratum 0 and 30 in stratum 1.

Table S3. Coding of categorical variables (Lactulose and Rifaximin) for the Cox regression analysis.

Categorical Variable Codings[a,c]			
		Frequency	(1)
Lactulose[b]	0	44	0
	1	76	1
Rifaximin[b]	0	57	0
	1	63	1

a. Category variable: Lactulose
b. Indicator Parameter Coding
c. Category variable: Rifaximin

For lactulose, “0” denotes absence of administration (n = 44) and “1” denotes administration (n = 76). For rifaximin, “0” denotes absence of administration (n = 111) and “1” denotes administration (n = 63). The coding uses an indicator parameter approach, with the non-administration category as the reference group in hazard ratio calculations.
